# Overcoming variant mutation-related impacts on viral sequencing and detection methodologies

**DOI:** 10.3389/fmed.2022.989913

**Published:** 2022-10-28

**Authors:** Yanxia Bei, Kaylinnette Pinet, Kyle B. Vrtis, Janine G. Borgaro, Luo Sun, Matthew Campbell, Lynne Apone, Bradley W. Langhorst, Nicole M. Nichols

**Affiliations:** New England Biolabs, Ipswich, MA, United States

**Keywords:** molecular diagnostics, NGS-next generation sequencing, infectious disease surveillance and control, SARS-CoV-2, COVID, bioinformatics, variants, viral genomics

## Abstract

Prompt and accurate pathogen identification, by diagnostics and sequencing, is an effective tool for tracking and potentially curbing pathogen spread. Targeted detection and amplification of viral genomes depends on annealing complementary oligonucleotides to genomic DNA or cDNA. However, genomic mutations that occur during viral evolution may perturb annealing, which can result in incomplete sequence coverage of the genome and/or false negative diagnostic test results. Herein, we demonstrate how to assess, test, and optimize sequencing and detection methodologies to attenuate the negative impact of mutations on genome targeting efficiency. This evaluation was conducted using *in vitro*-transcribed (IVT) RNA as well as RNA extracted from clinical SARS-CoV-2 variant samples, including the heavily mutated Omicron variant. Using SARS-CoV-2 as a current example, these results demonstrate how to maintain reliable targeted pathogen sequencing and how to evaluate detection methodologies as new variants emerge.

## Introduction

Global public health and research communities have rallied in an unprecedented manner to combat the COVID-19 pandemic. These efforts include extensive diagnostic testing to identify infected individuals and genomic sequencing to reveal the emergence of novel mutations within the SARS-CoV-2 RNA genome. These combined diagnostic and sequencing efforts helped the World Health Organization (WHO) classify populations of SARS-CoV-2 into variants based on shared characteristics such as genomic sequence and transmission dynamics (1).

**FIGURE 1:**
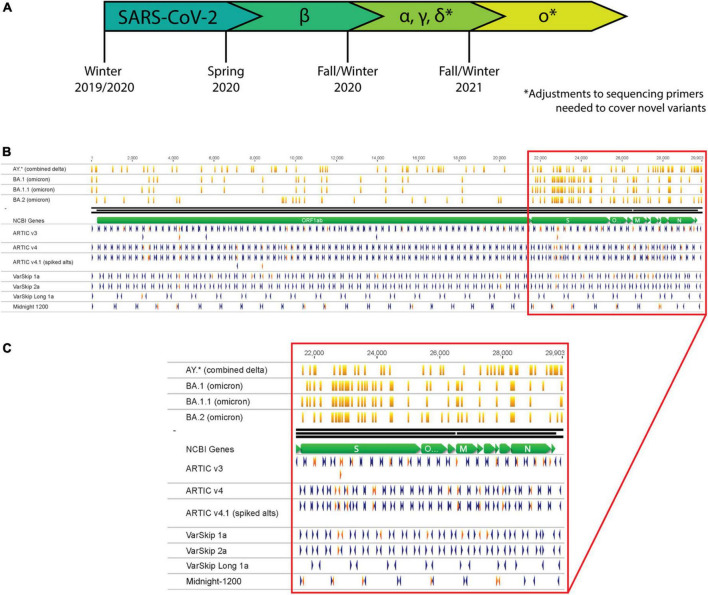
SARS-CoV-2 variants necessitate continual assessment of primers utilized for sequencing as variants overlap with primer sites. **(A)** Timeline representing the emergence of SARS-CoV-2 variants as determined by continual variant monitoring via sequencing. **(B)** Analysis of Delta and Omicron variants overlapping with primers from several SARS-CoV-2 primer schemes. Orange vertical lines mark mutation locations along variant strains. Blue and red arrowheads mark primer locations and orientations; red arrowheads are primers that overlap with mutation sites in one or more variant strains. **(C)** Zoom-in of primer-variant overlap analysis in the S gene to N gene region of the genome.

As early as Spring 2020, the global community began to take note of an increasing number of Covid-19 cases attributed to emerging SARS-CoV-2 variants (Figure 1A) (2, 3). The variants considered the greatest threat to global public health have been labeled as “Variants of Concern” (VOC) by the WHO, including Alpha, Beta, Gamma, Delta, and Omicron (1). The most widespread VOC thus far is Omicron, largely because it possesses genomic mutations that impart heightened transmissibility and immune-evasion relative to other SARS-CoV-2 variants (4–6). Public health authorities and researchers continue to utilize genomic sequencing to identify SARS-CoV-2 variants, track transmission chains, and assess the impact of novel mutations on current therapeutics or diagnostic assays.

The most widely applied and cost-effective SARS-CoV-2 sequencing methodologies use PCR to amplify overlapping segments across the genome. This approach was previously applied by the ARTIC Network for sequencing and monitoring Zika and Ebola, and then was quickly adapted for tracking SARS-CoV-2 (7). PCR requires annealing of primers to specific sites in the target genome, which can be disrupted by genetic mutations. Each of the most employed genomic sequencing primer schemes have been impacted by mutations acquired at primer target sites (Figure 1B). For genomic sequencing applications, failure to target and amplify the genome prior to library preparation results in amplicon loss (dropout), which generates gaps in genome coverage. For nucleic acid-based diagnostic assays, amplification failure could prevent viral detection and result in a false negative or inconclusive result. While sequencing and detection workflows can be designed to minimize the chances of a mutation disrupting their efficacy (8, 9), viruses continuously evolve and acquire mutations that necessitate re-assessment and optimization of these methodologies.

Herein, we demonstrate effective strategies to overcome genome mutation-related issues in sequencing and to evaluate diagnostic efficacy. Our evaluation of whole genome sequencing for a number of VOCs, using several common SARS-CoV-2 primer schemes identified several amplicon dropouts. We improved genomic coverage for VOCs by incorporating alternative SARS-CoV-2 primer schemes that were conscientiously designed to avoid targeting genomic sites with high mutation rates. The improved primer schemes include the VarSkip primers and more recent versions of ARTIC primer schemes. We also demonstrated how to evaluate the efficacy of a molecular diagnostic test that targets a site containing a mutation found in one VOC. Specifically, we assessed the impact of an Omicron mutation on the U.S. Centers for Disease Control and Prevention (CDC) 2019-nCoV panel, which has been granted Emergency Use Authorization (EUA) and incorporated into many diagnostic tests. Our assessment of two different amplification protocols concluded that 2019-nCoV target detection was unperturbed by the Omicron variant. These results demonstrate how to evaluate and overcome potential challenges from variant mutations to amplification-based methodologies.

## Results

### Mutations in SARS-CoV-2 variant genomes disrupt targeted whole-genome amplification

A complete genomic sequence is the ultimate resource for assessing potential impacts to sequencing and detection workflows. If the variant sequence is already determined, bioinformatic analysis can anticipate potential sequencing challenges by aligning the primer schemes to the known genomic sequence (Figures 1B,C). If mutations overlap with targeted priming sites, they may decrease amplification efficiency, resulting in amplicon dropouts and an incomplete genomic sequence.

**FIGURE 2:**
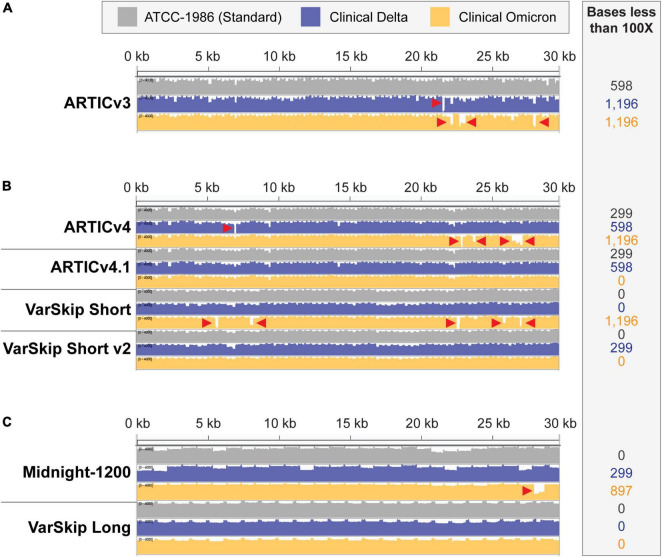
SARS-CoV-2 standard and variant genome coverage improves with multi-reference-based primer schemes. Integrative Genome Viewer visualization of read coverage across the SARS-CoV-2 genome (0–4,000 log scale). Genome coverage tracks for ATCC-1986 standard Wuhan-1 cultured RNA control templates in gray, clinical Delta sample templates in blue, and clinical Omicron sample templates in orange. Red arrowheads point to amplicon dropouts with < 100× coverage. The number of bases with less than 100× coverage are listed in the inset beside each genome coverage track. **(A)** Coverage of the SARS-CoV-2 genomes with ARTICv3 primers. **(B)** Coverage of SARS-CoV-2 genomes with ARTIC v4, ARTIC v4.1, VarSkip Short, and VarSkip Short v2 primers. **(C)** Coverage of SARS-CoV-2 genomes with Midnight-1200 and VarSkip Long primers.

Although bioinformatic analysis may reveal an overlap between a mutation and a target primer site, not all mutations will disrupt amplification. For example, mutations aligning with the 5’ end of a primer are less likely to inhibit amplification than mutations on the 3’ end. Consequently, a primer scheme should be experimentally tested to determine if mutations are detrimental and how significant their effects are. To demonstrate this point, we used the ARTIC V3 primer formulation included in the NEBNext ARTIC SARS-CoV-2 FS Library Prep Kit (E7658) to generate sequencing libraries for the Wuhan-1 cultured viral RNA and clinical RNA for Delta and Omicron SARS-CoV-2 variant samples. The ARTIC V3 primer scheme was designed by the ARTIC Network based on the original Wuhan-1 sequence and therefore provides complete coverage of that strain (10, 11) (Figure 2A). However, as the virus accumulated mutations, variant strains emerged and amplicon dropouts appeared (Figure 2A, red arrowheads). The Omicron variant contains the most mutations of any variant discovered to date, many of which coincide with sites targeted by primer sequences for amplicon PCR, and correspondingly, it also produces the most sequencing dropouts. Clinical RNA samples are not always readily available to a research laboratory. In these cases, commercially available synthetic or purified nucleic acids can be beneficial. Using these synthetic RNAs corresponding to the Beta, Gamma, and Delta variants, we were able to identify dropout sites where mutations disrupted amplification with the ARTIC V3 primer scheme (Supplementary Figure 1, red arrowheads). A limitation of using these RNA templates is that they consist of six separate RNA fragments and amplicons that bridge across the ends of the fragments will not amplify (Supplementary Figure 1, black arrowheads). As our data illustrate, regardless of sample types, clinical or synthetic RNA, primer sets need to be reevaluated and optimized to mitigate mutation-related impacts.

### Resilient pathogen-targeting primer design strategies improve genome coverage across variants

The success and utility of pathogen sequencing is principally measured in terms of genome coverage. In this study we compared genome coverage profiles for the initial SARS-CoV-2 strain, as well as the Delta and Omicron variants using several SARS-CoV-2 primer sets (Figure 2). The primer schemes we examined represent a range of primer set design strategies (Supplementary Table 1). For example, ARTIC v3 and Midnight-1200 primers are early single-reference genome primer sets, while ARTIC v4, ARTIC v4.1, and all VarSkip primers are multi-reference genome primer sets. Of note, the VarSkip Short and VarSkip Long primer schemes involved the use of more than a million reference sequences to avoid frequent mutations, whereas the ARTIC v4+ primers utilized a handful of VOC sequences circulating at the time of design to avoid specific mutations. A clear result seen through this study is that targeted pathogen sequencing benefits from the use of multiple references during initial primer scheme design. Our data show improved genome coverage across multiple samples and variants when comparing single reference primer schemes (i.e., ARTICv3 and Midnight-1200) and multi-reference primer schemes (i.e., ARTICv4.1, VarSkip Short v2, and VarSkip Long) (Figures 2A–C). However, multiple dropouts were seen with the initial multi-reference ARTICv4 and VarSkip Short primer schemes when sequencing an Omicron variant genome, due to the presence of several novel mutations in Omicron that were not prevalent in the references for those primer schemes (Figure 2B). Nevertheless, when multiple reference genomes for a pathogen are utilized to design primers against that genome, common variant sites can be avoided and thus a more resilient primer scheme design is generated.

We also found increased resilience to variants with the longer amplicon-based primer schemes (Midnight-1200 and VarSkip Long) in terms of a decreased likelihood for any given mutation to overlap with a primer binding site and therefore decreased probability of a variant-based amplicon dropout (Figures 1B,C, 2C). However, when a primer binding site in a long-amplicon primer set is negatively influenced by a variant, a larger gap in genome coverage results relative to the dropouts seen with shorter-amplicon primer schemes (Figures 2A–C). Thus, there is a cost-benefit analysis that must be considered when determining amplicon size in amplicon-based pathogen sequencing.

### Both primer spike-in and primer replacement strategies can be utilized to adjust primer schemes in response to novel variants

A carefully considered primer design can provide a degree of resilience to a primer scheme, however, no design is infallible. When variants do overlap with primer binding sites and primer efficiency assessments show a clear effect on genome coverage, primer schemes must be adjusted in response. The two most common approaches for altering primer schemes in response to novel variants include primer spike-ins and primer replacements. Both techniques require the design of new primers to avoid problematic variant sites and to rescue amplicon dropouts. The primer spike-in tactic is a fast, economic way to update an existing primer scheme, while the primer replacement approach requires more time and resources. The primer schemes examined in this study that represent these two separate strategies are the ARTICv4.1 and VarSkip Short v2 primers, respectively (Supplementary Table 1). Both primer scheme updates were designed to address the myriad of novel variant sites found in Omicron.

The ARTICv4.1 primer scheme published by the ARTIC network in December 2021 differs from the previous ARTIC v4 primer scheme by the inclusion of 11 additional primers. These 11 primers can be ordered individually and spiked into existing commercially available ARTICv4 primer mixes, with six primers spiked into Primer Mix 1 and five primers spiked into Primer Mix 2 (12–14). The VarSkip Short v2 primer scheme was published and made commercially available in February 2022. VarSkip Short v2 differs from the previous version by the replacement of 10 primers (15). Our data confirmed that both the ARTICv4.1 spiked-in primer mixes and VarSkip Short v2 updated primer mixes effectively rescued dropouts seen with an Omicron variant clinical sample (Figure 2B). Thus, both strategies can be successfully applied. Given that the spike-in tactic uses less resources, applying this approach for simple patches is practical, except in the case of large numbers of amplicon dropouts where the primer replacement approach is more appropriate.

### The SARS-CoV-2 Omicron variant contains a mutation overlapping with the centers for disease control and prevention N1 target

Variant mutations may also decrease the effectiveness of molecular diagnostic tests, which use quantitative PCR to amplify and detect nucleic acids. Quantitative PCR (qPCR) is the gold standard for diagnosing COVID-19 due to its high sensitivity and accuracy. In most qPCR diagnostic assays, the amplification signal is detected in real time using a fluorescent probe. The probe anneals to the target amplicon and the fluorophore is released by DNA polymerase cleavage of the probe. Consequently, these tests can be sensitive to variant mutations since both amplification and detection require efficient annealing of oligonucleotides to target sequences. It is therefore essential to monitor the emergence of variants and to evaluate the impact of the mutations on virus detection to avoid false negative results.

**FIGURE 3:**
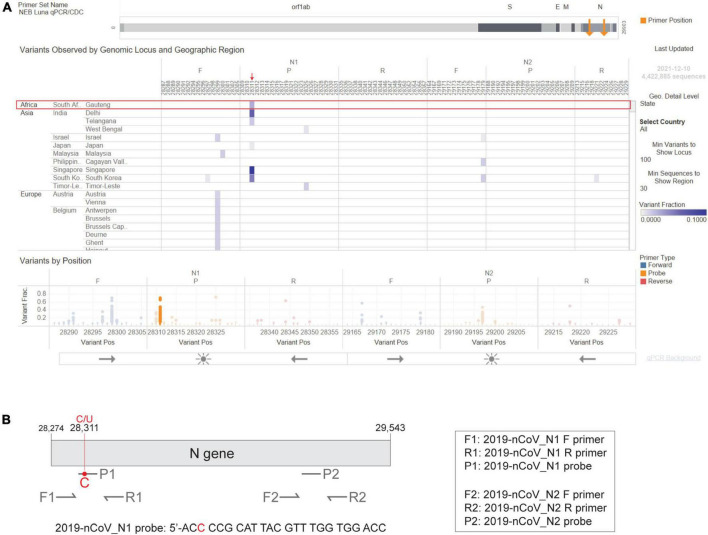
The Omicron N gene contains a mutation targeted by the 2019-nCoV_N1 detection probe. The CDC 2019-Novel Coronavirus (2019-nCoV) Real-Time RT-PCR Diagnostic Panel includes two primer-probe sets that target the SARS-CoV-2 N gene, named 2019-nCoV_N1 and 2019-nCoV_N2. **(A)** Visual depiction of Omicron variant mutation within the N1 probe site generated using the Primer Monitor online tool (Tableau worksheet exported from Primer Monitor tool, primer set name “NEB Luna qPCR/CDC”). The Omicron variant from Gauteng, South Africa and the mutation are annotated with a red box and an arrow, respectively. **(B)** Schematic representation of the two CDC primer-probe sets. Each set includes one forward primer (F), one reverse primer (R), and one fluorescent probe (P). The SARS-CoV-2 Omicron variant has a C to U mutation at position 28,311, which is within the 2019-nCoV_N1 probe (P1) target sequence. Not drawn to scale.

In February 2020, the CDC released a qPCR-based laboratory test called the CDC 2019-Novel Coronavirus (2019-nCoV) Real-Time RT-PCR Diagnostic Panel, which targets two sites on the SARS-CoV-2 Nucleocapsid (N) gene, namely 2019-nCoV_N1 and 2019-nCoV_N2. The CDC SARS-CoV-2 assay targets have been granted EUA and have subsequently been incorporated in various SARS-CoV-2 diagnostic assays. As the Omicron variant emerged, we used the NEB Primer Monitor tool^1^ to assess the potential impact of its mutations on the CDC 2019-nCoV panel. The Primer Monitor tool revealed a C to U mutation at genomic position 28,311, which corresponds to the 3rd nucleotide from the 5’ end of the 2019-nCoV_N1 probe target sequence (Figures 3A,B). Given the location of this mismatch, it is unlikely that the CDC 2019-nCoV_N1 target would fail completely but impacts to assay sensitivity cannot be dismissed. Below we describe general strategies for quickly testing the impact of variant mutations on qPCR-based detection.

### Quantitative PCR detection using RNA extracted from *in vitro*-transcribed RNA input

One strategy for evaluating SARS-CoV-2 detection assays is to use commercially available templates by purchasing synthetic DNA (to prepare RNA) or RNA, but the synthesis and delivery of the DNA/RNA may delay testing by weeks. Alternatively, one could use IVT RNA as the input template for evaluating detection assays. This is ideal when a clinical sample of a new variant is not available, and the evaluation is time sensitive. In fact, preparation of IVT RNA containing the mutation of interest can be completed within 1 week (Supplementary Figure 2A). The IVT RNA strategy requires a plasmid containing the target sequence with a T7 promoter, such as the SARS-CoV-2 Positive Control plasmid (N2117S) that contains the full N gene (GenBank MN908947.3). To generate an RNA template to evaluate the Omicron mutation’s impact on CDC 2019-nCoV_N1 detection efficiency, the appropriate Omicron variant mutation was incorporated into the N gene sequence using the Q5 Site-Directed Mutagenesis Kit. Mutant and wild-type RNA templates were subsequently generated by T7 RNA Polymerase to test whether the Omicron mutation at position 28,311 disrupted qPCR efficacy (Supplementary Figure 2B). Correct size and high purity were confirmed via agarose gel electrophoresis (Supplementary Figure 2C).

**FIGURE 4:**
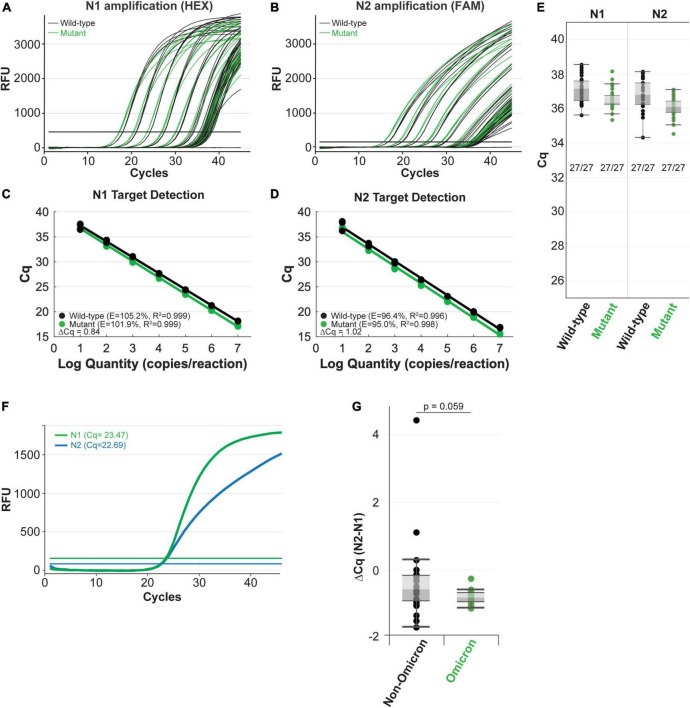
The NEB SARS-CoV-2 multiplex assay efficiently detects N gene IVT RNA carrying the Omicron mutation **(A–E)** and clinical Omicron RNA **(F,G)** with the CDC 2019-nCoV_N1 primer-probe set. Amplification efficiency in panels **(A–D)** was evaluated in triplicate over a 7-log range (10^7^-10 copies/reaction) of synthetic N gene RNA for wild-type (black) vs. the Omicron variant (green). Detection sensitivity **(E)** was evaluated with 10 copies of RNA per reaction with 27 replicates per condition. All reactions tested with 10 copies of input RNA were detected by qPCR. Both the N1 (green) and N2 (blue) targets were efficiently amplified from clinical Omicron RNA samples using the NEB Luna SARS-CoV-2 RT-qPCR Multiplex Assay Kit, which includes the CDC 2019-nCoV_N1 and 2019-nCoV_N2 primer-probe sets **(F,G)**. Cq values for each clinical sample shown **(G)** are included in Supplementary Table 2.

Next, we compared amplification of the mutant RNA template to the wild-type N gene RNA template using the NEB SARS-CoV-2 RT-qPCR Multiplex Assay Kit (E3019). This assay kit simultaneously detects the N1 (HEX), N2 (FAM), and human RNase P (Cy5) targets. The N1 and N2 targets were amplified efficiently across a 7-log dilution series (10^7^-10 copies/reaction) of mutant and wild-type input RNAs (Figures 4A,B). In these experiments, the N2 primer-probe set can also serve as an internal control to correct minor differences in RNA template input because the Omicron variant does not have a mutation within the region targeted by the N2 primers or the probe (Figure 3A). The mutant RNA crossed threshold ∼1 cycle faster than wild-type RNA, likely due to slightly higher input amount. After correcting the RNA input amount based on the N2 target, there is less than a 0.2 difference in the average delta C_*q*_ for the N1 target amplification between the mutant and wild-type RNA (Figures 4C,D). This is well within normal day-to-day and user-to-user variation and suggests equivalence in amplification speed. Importantly, the mutation did not decrease assay sensitivity as 27 out of 27 reactions with low RNA copy number (10 copies per reaction) were detected for the wild-type and mutant RNA with both the N1 and N2 targets (Figure 4E). We also checked amplification efficiency using the SalivaDirect workflow. SalivaDirect is a simplified, non-invasive, and flexible SARS-CoV-2 diagnostic platform granted EUA by the FDA (16). It uses the CDC 2019-nCoV_N1 primer-probe set as the only detection target for SARS-CoV-2 along with human RNase P as an internal control. Amplification using the SalivaDirect conditions was unimpeded by the N1 Omicron mutation (Supplementary Figure 3). Consequently, we conclude that both the NEB SARS-CoV-2 Multiplex Assay Kit and the SalivaDirect amplification conditions can reliably detect the Omicron N gene using the CDC 2019-nCoV_N1 primer-probe set.

### Quantitative PCR detection using RNA extracted from clinical samples

When possible, it is also important to determine whether the variant mutations impact detection of clinical samples. In contrast to the IVT RNA approach, RNA extracted from the variant virus has the added benefit that it includes all the genomic mutations. We validated the IVT RNA results described above using Omicron viral RNA extracted from clinical samples. Using the SARS-CoV-2 Multiplex RT-qPCR Assay Kit, both the N1 and N2 targets were efficiently detected from Omicron RNA (example from a single sample shown in Figure 4F). Cq values will vary among clinical samples due to differing amounts of input RNA (viral load will vary from patient to patient and day-to-day during an infection). To account for these differences, we compared the N1 target Cq relative to the N2 target, which does not contain a mutation in the Omicron variant, for each sample. We also compared N1 and N2 target detection from non-Omicron (that do not contain mutations targeted by this assay) and Omicron clinical samples. To evaluate the detection speed for the N1 target, we calculated the delta Cq between N2 and N1 for each sample (Supplementary Table 2 and Figure 4G). The difference in detection speed within each sample were comparable between the non-Omicron and the Omicron groups, suggesting N1 target detection is not compromised in the Omicron clinical samples.

## Discussion

All viruses mutate and evolve over time, which necessitates regular reevaluation of sequencing and detection workflows to sustain their accuracy and reliability. Our results describe how to conduct these evaluations using either commercially available control RNA templates or RNA extracted from clinical SARS-COV-2 samples, and we demonstrate how to generate RNA templates for diagnostic evaluations in a timely manner when neither of the two options are available. While these results focused on reacting to emerging SARS-CoV-2 variants, the targeted sequencing and diagnostic assay approaches highlighted here are generally applicable to other pathogens and organisms.

Widespread application of genomic sequencing has become an essential epidemiological tool for monitoring and controlling the spread of SARS-CoV-2. Sequencing reveals the mutational signature of the virus, which not only identifies the type of variant but also informs about potential impacts to therapies and diagnostic assays. Consequently, sequencing approaches are optimized toward maximizing genome coverage. Careful design of overlapping amplicon primer schemes helps to mitigate the potential impact of acquired mutations on genome sequencing coverage. When a mutation causes an amplicon dropout to occur, the primer set must be reevaluated and optimized. Our evaluation of five different primer sets concluded that the NEB VarSkip Long primer set achieved the greatest sequence coverage, with respect to Omicron sequencing despite the drastic increase and unique pattern of mutations seen in this variant. The advantage seen with the VarSkip Long primer set in this case, can be attributed to the primer scheme design approach. This VarSkip Long design approach minimized the number of overall primer sites and is based on over one million SARS-CoV-2 reference genome sequences to avoid the more frequently mutated genome sites. However, if RNA templates are degraded or additional genome variants arise that negatively impact VarSkip Long primer binding, then the length of even a single amplicon dropout would impact more of the genome than multiple amplicon dropouts seen with a primer scheme based on short amplicons. In practice, VarSkip Short v2’s balance of amplicon length (∼550 bp) and effectiveness on a range of sample types has proven to be the most effective in our hands. To obtain complete coverage of unusual genomes, access to multiple primer schemes has proven essential. Once sufficient genomic coverage is obtained, researchers can assess and respond to viral genome mutations that could impact therapeutics or diagnostic assays.

The U.S. Food and Drug Administration issued guidelines for evaluating the impact of variants on COVID-19 detection tests (17). These guidelines breakdown important considerations for both design and testing of molecular diagnostic assays. Importantly, molecular assays should be designed to target the genomes of the currently circulating variants and to include redundancy to help maintain performance as future variants emerge. Redundancy is built into the assays by targeting multiple sites within the genome. This strategy proved to be important for detection of several of the SARS-CoV-2 variants, including Alpha and Omicron. When these variants first emerged, several commonly used PCR tests failed to detect the heavily mutated S gene (called S gene dropout). However, because many of these assays also targeted other sites, the virus was still detected, and the S gene dropout signature was used to help track variant progression. The Omicron genome also contained an N gene mutation corresponding to the CDC-designed N1 target probe sequence in the 2019-nCoV panel, which has been incorporated into many diagnostic assays. Fortunately, our evaluation of the performances of two different amplification conditions found the N1 site is efficiently detected with the CDC 2019-nCoV_N1 primer-probe set, despite the target probe sequence mutation. Our evaluation has determined that the mutation does not diminish amplification performance using the CDC 2019-nCoV_N1 primer-probe set in our optimized conditions using purified RNA; however, wastewater surveillance work published in a medRxiv preprint suggests the mutation alters the N1 probe binding efficiency during ddPCR (18). Interestingly, the ddPCR approach used the same CDC 2019-nCoV-N1 primer-probe set. It is therefore essential that each assay workflow is evaluated for variant detection efficacy if the primer-probe set harbors a mutation. Once a mutation is detected within a testing target, our IVT RNA approach described herein can be utilized to evaluate the diagnostic test efficacy in a timely manner. Furthermore, this strategy can be applied to other nucleic acid based diagnostic tests, including LAMP (19).

As more variants emerge, it is essential that developers continuously track new mutations and assess their potential impact on sequencing, therapeutic, and diagnostic assays. This process includes aligning the primer/probe sequences against genomes found in limited-access databases such as GISAID or public INSDC resources (e.g., Genbank), to evaluate whether novel mutations may impact the assays. The NEB Primer Monitor tool (see footnote text 1) can assist in identifying potentially problematic mutations by continually monitoring registered primer-probe sets for overlapping mutations (20). While the Primer Monitor has been implemented with a simple reference alignment approach, graph genomes have been effectively used to represent other complex populations (21). SARS-CoV-2 mutation patterns have mostly resulted in short deletions and substitutions, so graph genome techniques have not been required. Future variants with more divergence from the NC_045512 reference sequence might benefit from a more sophisticated graph genome approach. As previously noted, the tool revealed an overlap between an Omicron mutation at position 28,311 and the CDC 2019-nCoV_N1 probe target sequence (Figure 3A). We have also used the Primer Monitor tool to evaluate other mutations in SARS-CoV-2 variants overlapping with the CDC 2019-nCoV panel. After testing, we found that some of these mutations decreased assay sensitivity (20), whereas others, such as the AY43 (Delta) variant, did not impact N gene assay sensitivity (Supplementary Figure 4). We also determined that N gene assay sensitivity was unaffected by the mutations contained in a new Omicron variant (BA.5, data not shown). Tracking the emergence of new mutations and testing the performance of primer-probe sets with the variant sequences by qPCR will help ensure the continued reliability of COVID-19 diagnostic tests.

### Methods

#### Clinical delta and omicron RNA collection and extraction

For clinical samples, RNA extractions were performed as described (22) following NEB’s protocol for RNA extraction from saliva utilizing the Monarch Total RNA Miniprep Kit (T2010). The final total RNA was eluted in either 50 or 100 μl of nuclease free water prior to storage at –20°C for less than 1 week or –80°C for long term storage.

#### SARS-CoV-2 genomic sequencing

The NEBNext ARTIC SARS-CoV-2 FS Library Prep Kit (E7658) and workflow were followed to generate targeted amplicons and sequencing libraries. Either deidentified clinical samples or commercially available RNA controls were utilized as templates for cDNA synthesis and amplification. For commercially available templates, one-thousand genomic copies of standard or variant SARS-CoV-2 viral gRNA controls (ATCC^®^ VR-1986 from ATCC; SARS-CoV-2 RNA Control 16, 17, 18, and 23 from Twist Bioscience, representing the Beta, Gamma, Kappa, and Delta variants, respectively) in 100 ng of Universal Human Reference RNA (ThermoFisher^®^ QS0639) were used. For deidentified clinical samples, no Universal Human Reference RNA was added, and equal input volumes were used for cDNA synthesis and amplification across the various targeted amplification reactions, regardless of the targeting primer set. Amplicons were generated using either the NEBNext ARTIC SARS-CoV-2 primer pools (ARTICv3), NEBNext VarSkip Short SARS-CoV-2 primer pools, ARTICv4 primer pools, or IDT xGEN SARS-CoV-2 Midnight-1200 Amplicon Panel (10007184). Libraries were constructed using the NEBNext ARTIC SARS-CoV-2 FS Library Prep Kit (Illumina) and sequenced on a MiSeq^®^ instrument (2 × 75 bp). The Galaxy IWC SARS-CoV-2 reference based assembly workflow was used to produce consensus sequences and calculate coverage depth per base (23–47).

#### Bioinformatic analysis of primer-variant overlaps

Bioinformatic analysis of primer-variant overlaps followed procedures outlined on the Primer Monitor Tool webpage. Publicly available sequences for each viral lineage were aligned to the NC_045512.2 reference sequence [minimap2 –r 10,000 –score-N = 0, 2.17 (39), samtools 1.11 (31, 32, 43)]. Variants > 2% frequency (freebayes 2.2.0) (44) were evaluated for overlap with primer regions (bedtools (Version 2.29.2) (42) and displayed (Geneious Prime 2021.0.3). Due to the rapid increase in omicron sequences, BA.1, BA.2, BA.4, and BA.5 lineage variants were identified by classifying recent consensus sequences submitted to GISAID using pangolin—usher (Version 4.0.5) (28) before alignment (minimap 2.2) (39) and variant calling (2% frequency threshold). Variants were manually filtered to remove Delta contamination. Exclusion criteria: Variants present in Omicron lineage sequences and Delta lineage sequences that dropped in frequency between November 2021 and January 2022 were excluded.

#### Preparation of *in vitro*-transcribed RNA

To generate the SARS-CoV-2 N gene RNA harboring the C to U mutation at position 28,311, site-directed mutagenesis (Q5 Site-Directed Mutagenesis Kit, NEB E0554S) was performed using the SARS-CoV-2 Positive Control (N gene) plasmid (NEB N2117) as a template, and the mutation was confirmed by Sanger Sequencing. The wild-type and mutant N gene RNA was subsequently synthesized by *in vitro* transcription (HiScribe T7 High Yield Synthesis Kit, NEB E2040) from linearized plasmids containing either wild-type or mutant N genes, respectively. The resulting RNA was purified using the Monarch RNA Cleanup Kit (NEB, E2050) and quantitated with the Qubit RNA BR Assay Kit (ThermoFisher Scientific, Q33224) to calculate the RNA copy number. The purity and quality of the plasmids and RNA were assessed on a 1.2% agarose gel, electrophoresed for 1 h in 0.5x Tris-Borate-EDTA Buffer.

#### RT-quantitative PCR amplification of *in vitro*-transcribed RNA

To evaluate the impact of the 28,311 C to U mutation on the 2019-nCov_N1 target detection, a 7-log dilution series (10^7^–10 copies/reaction) was prepared for both the wild-type and the mutant RNA, with 10 ng of Jurkat total RNA (BioChain, R1255815-50) included as an internal control. The mutant and wild-type target RNA were subsequently amplified using either the NEB Luna SARS-CoV-2 RT-qPCR Multiplex Assay Kit (E3019) or the Luna Universal Probe One-Step RT-qPCR Kit (NEB E3006) following the SalivaDirect RT-qPCR amplification protocol (16). For sensitivity evaluations, 27 reactions containing 10 copies/reaction were performed using either wild-type or mutant RNA. Briefly, for the Luna SARS-CoV-2 RT-qPCR assay, the N1 (HEX), N2 (FAM), and RP (Cy5) targets were simultaneously detected using the following cycling conditions: carryover prevention (25°C for 30 s), cDNA synthesis (55°C for 10 min), initial denaturation (95°C for 1 min) and 45 cycles of denaturation (95°C for 10 s) and annealing/elongation (60°C for 30 s) plus a plate read step. For the SalivaDirect RT-qPCR, the Luna Universal Probe One-Step RT-qPCR Kit (NEB E3006) was used to detect the N1 (FAM) and the RP (Cy5) targets simultaneously using the following cycling conditions: cDNA synthesis step (52°C for 10 min), initial denaturation (95°C for 2 min) and 45 cycles of denaturation (95°C for 10 s) and annealing/elongation (55°C for 30 s) plus a plate read step. The qPCR data was collected on a Bio-Rad CFX96 qPCR instrument (96-well, 20 μl reactions).

#### RT-quantitative PCR amplification of RNA extracted from clinical Omicron specimens

For the clinical sample, 2 μL of the extracted RNA was used in the Luna SARS-CoV-2 RT-qPCR assay as described above, and the data was collected on a Bio-Rad CFX96 qPCR instrument (96-well).

## Data availability statement

The datasets presented in this study can be found in online repositories. The names of the repository and accession numbers can be found in the article/Supplementary material.

## Ethics statement

The studies involving human participants were reviewed and approved by WCG IRB (study #1340251). The patients/participants provided their written informed consent to participate in this study.

## Author contributions

YB, KV, KP, JB, LA, BL, and NN conceptualized and designed the experiments. YB, KP, LS, and JB performed the experiments. MC and BL develop and maintain the Primer Monitor tool. NN supervised the work. KV, YB, KP, and NN wrote the manuscript with input from the other co-authors. All authors contributed to the article and approved the submitted version.

## Footnotes

1
https://primer-monitor.neb.com

## References

[1] World Health Organization[WHO]. *Tracking SARS-CoV-2 variants.* (2020). Available online at: https://www.who.int/en/activities/tracking-SARS-CoV-2-variants/ (accessed April 20, 2022).

[2] Choi JY Smith DM. SARS-CoV-2 variants of concern. *Yonsei Med J.* (2021) 62:961–8. 10.3349/YMJ.2021.62.11.961 34672129 PMC8542474

[3] Gisaid - NextStrain. *Genomic epidemiology of SARS-CoV-2 with subsampling focused globally over the past 6 months.* (2022). Available online at: https://www.gisaid.org/phylodynamics/global/nextstrain/ (accessed April 21, 2022).

[4] Ferguson N Ghani A Cori A Hogan A Hinsley W. *Report 49 - growth, population distribution and immune escape of omicron in England.* (2021). Available online at: https://www.imperial.ac.uk/mrc-global-infectious-disease-analysis/covid-19/report-49-Omicron/ (Accessed April 20, 2022).

[5] McCallum M Czudnochowski N Rosen LE Zepeda SK Bowen JE Walls AC Structural basis of SARS-CoV-2 omicron immune evasion and receptor engagement. *Science.* (2022) 375:894–8. 10.1126/SCIENCE.ABN8652/SUPPL_FILE/SCIENCE.ABN8652_SM.PDF 35076256 PMC9427005

[6] Ao D Lan T He X Liu J Chen L Baptista-Hon DT CoV-2 omicron variant: *Immune escape* and vaccine development. *MedComm.* (2022) 3:e126. 10.1002/MCO2.126 35317190 PMC8925644

[7] Quick J Grubaugh ND Pullan ST Claro IM Smith AD Gangavarapu K Multiplex PCR method for MinION and *Illumina sequencing* of zika and other virus genomes directly from clinical samples. *Nat Proto.* (2017) 12:1261–76. 10.1038/nprot.2017.066 28538739 PMC5902022

[8] Tyson JR James P Stoddart D Sparks N Wickenhagen A Hall G Improvements to the ARTIC multiplex PCR method for SARS-CoV-2 genome sequencing using nanopore. *bioRxiv* [preprint] (2020). 10.1101/2020.09.04.283077 32908977 PMC7480024

[9] Davis JJ Long SW Christensen PA Olsen RJ Olson R Shukla M Analysis of the ARTIC version 3 and version 4 SARS-CoV-2 primers and their impact on the detection of the G142D amino acid substitution in the spike protein. *Microbiol Spectr.* (2021) 9:e0180321. 10.1128/Spectrum.01803-21 34878296 PMC8653831

[10] Zenodo. *artic-network/primer-schemes: v1.1.1.* (2022). Available online at: https://zenodo.org/record/4020380#.YmGuWvPMLZU (accessed April 20, 2022).

[11] Quick J. *nCoV-2019 sequencing protocol.* (2022). Available online at: https://www.protocols.io/view/ncov-2019-sequencing-protocol-bp2l6n26rgqe/v1?version_warning=no (accessed April 20, 2022).

[12] Sars-CoV. *SARS-CoV-2 version 4 scheme release - laboratory - ARTIC real-time genomic surveillance.* (2022). Available online at: https://community.artic.network/t/sars-cov-2-version-4-scheme-release/312 (accessed April 20, 2022).

[13] Sars-CoV. *SARS-CoV-2 V4.1 update for omicron variant - laboratory - ARTIC real-time genomic surveillance.* (2022). Available online at: https://community.artic.network/t/sars-cov-2-v4-1-update-for-omicron-variant/342 (accessed April 20, 2022).

[14] Lambisia AW Mohammed KS Makori TO Ndwiga L Mburu MW Morobe JM Optimization of the SARS-CoV-2 ARTIC network V4 primers and whole genome sequencing protocol. *Front Med.* (2022) 9:322. 10.3389/FMED.2022.836728/BIBTEX PMC8891481 35252269

[15] GitHub - nebiolabs/VarSkip. *VarSkip multiplex PCR designs for SARS-CoV-2 sequencing.* (2022). Available online at: https://github.com/nebiolabs/VarSkip (accessed May 18, 2022).

[16] Vogels CBF Watkins AE Harden CA Brackney DE Shafer J Wang J SalivaDirect: a simplified and flexible platform to enhance SARS-CoV-2 testing capacity. *Med.* (2021) 2:263–80. 10.1016/J.MEDJ.2020.12.010 33521748 PMC7836249

[17] FDA. *Policy for evaluating impact of viral mutations on COVID-19 tests.* (2022). Available online at: https://www.fda.gov/regulatory-information/search-fda-guidance-documents/policy-evaluating-impact-viral-mutations-covid-19-tests (accessed April 20, 2022).

[18] Schussman MK Roguet A Schmoldt A Dinan B McLellan SL. Wastewater surveillance using ddPCR reveals highly accurate tracking of omicron variant due to altered N1 probe binding efficiency. *medRxiv* [preprint] (2022). 10.1101/2022.02.18.22271188

[19] Patton GC Alpaslan E Ren G Zhang Y Tanner NA Nichols NM. *Optimizing a rapid, isothermal workflow for detection of SARS-CoV-2 viral RNA using WarmStart.* New England: LAMP Reagents with UDG (2021).

[20] Campbell M Bei Y Nichols N Langhorst B. *Primer monitor: an online tool to track SARS-CoV-2 variants that may impact primers used in diagnostic assays.* (2021). Available online at: https://www.neb.com/-/media/nebus/files/application-notes/whitepaper_primer_monitor_an_online_tool_to_track_sars-cov-2_variants.pdf?rev=57600d457f244447aa65eeca56042498. (accessed December 20, 2021).

[21] Paten B Novak AM Eizenga JM Garrison E. Genome graphs and the evolution of genome inference. *Genome Res.* (2017) 27:665–76. 10.1101/GR.214155.116 28360232 PMC5411762

[22] Li Z Bruce JL Cohen B Cunningham C Jack WE Kunin K Development and implementation of a simple and rapid extraction-free saliva SARS-CoV-2 RT-LAMP workflow for workplace surveillance. *PLoS One.* (2022) 17:e0268692. 10.1371/JOURNAL.PONE.0268692 35617204 PMC9135294

[23] Wilm A Aw PPK Bertrand D Yeo GHT Ong SH Wong CH LoFreq: a sequence-quality aware, ultra-sensitive variant caller for uncovering cell-population heterogeneity from high-throughput sequencing datasets. *Nucleic Acids Res.* (2012) 40:11189–201. 10.1093/NAR/GKS918 23066108 PMC3526318

[24] García-Alcalde F Okonechnikov K Carbonell J Cruz LM Götz S Tarazona S Qualimap: evaluating next-generation sequencing alignment data. *Bioinformatics.* (2012) 28:2678–9. 10.1093/BIOINFORMATICS/BTS503 22914218

[25] Okonechnikov K Conesa A García-Alcalde F. Qualimap 2: advanced multi-sample quality control for high-throughput sequencing data. *Bioinformatics.* (2016) 32:292–4. 10.1093/BIOINFORMATICS/BTV566 26428292 PMC4708105

[26] Ewels P Magnusson M Lundin S Käller M. MultiQC: summarize analysis results for multiple tools and samples in a single report. *Bioinformatics.* (2016) 32:3047–8. 10.1093/BIOINFORMATICS/BTW354 27312411 PMC5039924

[27] Grubaugh ND Gangavarapu K Quick J Matteson NL de Jesus JG Main BJ An amplicon-based sequencing framework for accurately measuring intrahost virus diversity using PrimalSeq and iVar. *Genome Biol.* (2019) 20:1–19. 10.1186/S13059-018-1618-7/FIGURES/9 30621750 PMC6325816

[28] O’Toole Á Scher E Underwood A Jackson B Hill V McCrone JT Assignment of epidemiological lineages in an emerging pandemic using the pangolin tool. *Virus Evol.* (2021) 7:64. 10.1093/VE/VEAB064 34527285 PMC8344591

[29] GitHub - Nanoporetech/Medaka. *Sequence correction provided by ONT Research.* (2022). Available online at: https://github.com/nanoporetech/medaka (accessed September 22, 2022).

[30] Cingolani P Patel VM Coon M Nguyen T Land SJ Ruden DM Using *Drosophila melanogaster* as a model for genotoxic chemical mutational studies with a new program, SnpSift. *Front Genet.* (2012) 3:35. 10.3389/FGENE.2012.00035/BIBTEX PMC3304048 22435069

[31] GitHub - Samtools. *Tools (written in C using htslib) for manipulating next-generation sequencing data.* (2022). Available online at: https://github.com/samtools/samtools (accessed September 22, 2022).

[32] Li H. *Mathematical Notes on Samtools Algorithms*. (2010). Available online at: http://lh3lh3.users.sourceforge.net/download/samtools.pdf (accessed April 20, 2022).

[33] Durbin H. *Segregation based Metric For Variant Call QC*. (2014). Available online at: http://samtools.github.io/bcftools/rd-SegBias.pdf (accessed April 20, 2022).

[34] Danecek P Schiffels S Durbin R. *Multiallelic Calling Model in Bcftools (−m)*. (2016). Available online at: https://samtools.github.io/bcftools/call-m.pdf (accessed April 20, 2022).

[35] Li H Barrett J. A statistical framework for SNP calling, mutation discovery, association mapping and population genetical parameter estimation from sequencing data. *Bioinformatics.* (2011) 27:2987–93. 10.1093/BIOINFORMATICS/BTR509 21903627 PMC3198575

[36] Li H. Improving SNP discovery by base alignment quality. *Bioinformatics.* (2011) 27:1157–8. 10.1093/BIOINFORMATICS/BTR076 21320865 PMC3072548

[37] HTS format specifications. *SAM/BAM and related specifications.* (2022). Available online at: https://samtools.github.io/hts-specs/ (accessed September 22, 2022).

[38] Cingolani P Platts A Wang LL Coon M Nguyen T Wang L A program for annotating and predicting the effects of single nucleotide polymorphisms, SnpEff: SNPs in the genome of *Drosophila melanogaster* strain w1118; iso-2; iso-3. *Fly.* (2012) 6:80–92. 10.4161/FLY.19695/SUPPL_FILE/KFLY_A_10919695_SM0001.ZIP 22728672 PMC3679285

[39] Li H. Minimap2: pairwise alignment for nucleotide sequences. *Bioinformatics.* (2018) 34:3094–100. 10.1093/BIOINFORMATICS/BTY191 29750242 PMC6137996

[40] Li H Durbin R. Fast and accurate long-read alignment with burrows–wheeler transform. *Bioinformatics.* (2010) 26:589–95. 10.1093/BIOINFORMATICS/BTP698 20080505 PMC2828108

[41] Li H Durbin R. Fast and accurate short read alignment with burrows–wheeler transform. *Bioinformatics.* (2009) 25:1754–60. 10.1093/BIOINFORMATICS/BTP324 19451168 PMC2705234

[42] Quinlan AR Hall IM. BEDTools: a flexible suite of utilities for comparing genomic features. *Bioinformatics.* (2010) 26:841–2. 10.1093/BIOINFORMATICS/BTQ033 20110278 PMC2832824

[43] Li H Handsaker B Wysoker A Fennell T Ruan J Homer N The sequence alignment/map format and SAMtools. *Bioinformatics.* (2009) 25:2078–9. 10.1093/BIOINFORMATICS/BTP352 19505943 PMC2723002

[44] Garrison E Marth G. Haplotype-based variant detection from short-read sequencing. *Quant Biol.* (2012) 1207:3907. 10.48550/arxiv.1207.3907 35895330

[45] Chen S Zhou Y Chen Y Gu J. fastp: an ultra-fast all-in-one FASTQ preprocessor. *Bioinformatics.* (2018) 34:i884–90. 10.1093/BIOINFORMATICS/BTY560 30423086 PMC6129281

[46] Aksamentov I Roemer C Hodcroft EB Neher RA. Nextclade: clade assignment, mutation calling and quality control for viral genomes. *J Open Source Softw.* (2021) 6:3773. 10.21105/JOSS.03773

[47] Maier W. *Sars-Cov-2-Pe-Illumina-Artic-Variant-Calling/COVID-19-PE-ARTIC-Illunina*. (2022). Available online at: https://workflowhub.eu/workflows/110 (accessed August 20, 2022).

[48] Bei Y Vrtis KB Borgaro JG Langhorst BW Nichols NM. The omicron variant mutation at position 28,311 in the SARS-CoV-2 N gene does not perturb CDC N1 target detection. *medRxiv* [preprint] (2021). 10.1101/2021.12.16.21267734

