# Supplementary material for: Overcoming variant mutation-related impacts on viral sequencing and detection methodologies
Source: Front Med (Lausanne). 2022 Oct 28;9:989913. doi: 10.3389/fmed.2022.989913 (PMC9650041; doi:10.3389/fmed.2022.989913)
Supplement: Supplementary file 1 [file Data_Sheet_1.PDF]

Supplementary Material

Supplementary Material

Supplementary Figures and Tables

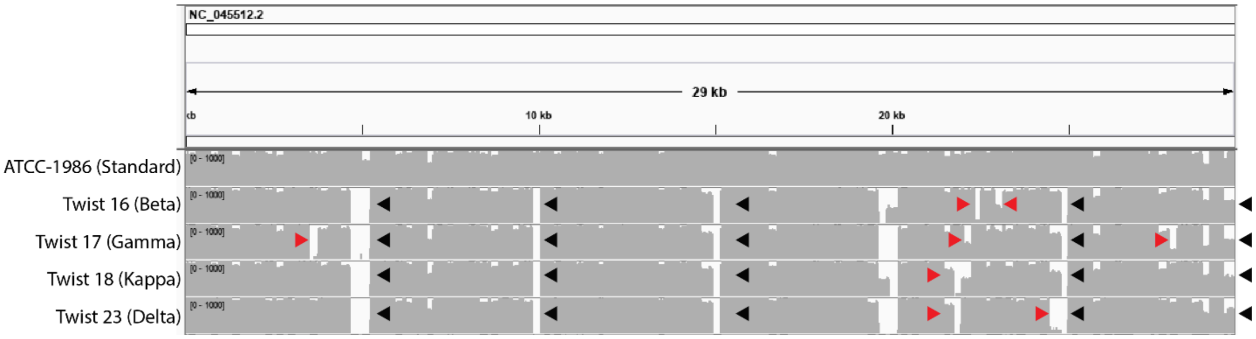

Supplemental Fig. 1. ARTICv3 genome coverage assessed with synthetic Twist control variant templates. Integrative Genome Viewer visualization of read coverage across the SARS-CoV-2 genome (0-1000 log scale). Genome coverage tracks for ATCC-1986 standard Wuhan-1 cultured RNA control template and synthetic variant templates for Beta, Gamma, Kappa, and Delta variants. Black arrowheads point to drops in genome coverage due to synthetic breaks in control templates. Red arrowheads point to amplicon dropouts due to variant sequence effects on primer binding.

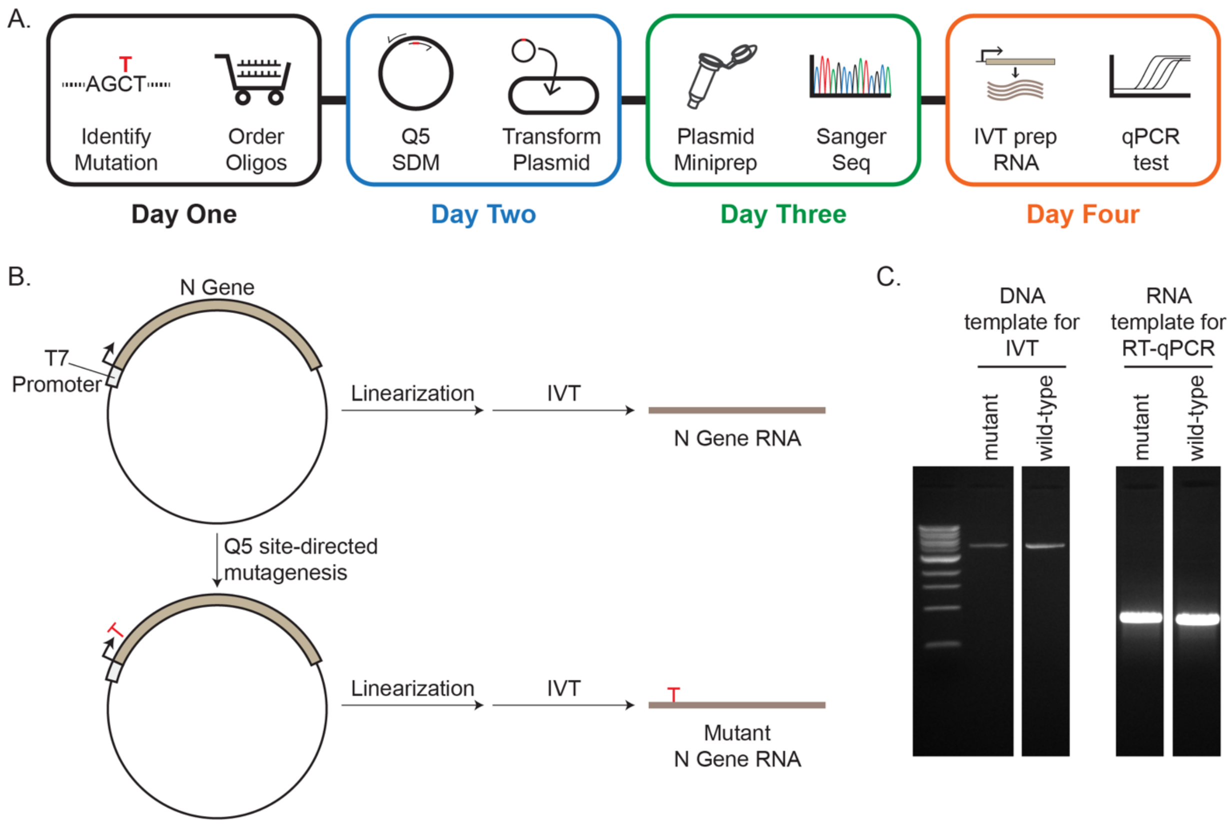

Supplemental Fig. 2. A scheme to generate IVT RNA templates to evaluate the impact of mutations on target detection (A and B). The quality of the linearized DNA plasmids used as templates for in vitro transcription (IVT) and the RNA yielded from IVT were assessed on a 1.2% agarose gel, run for 1 h in 0.5x Tris-Borate-EDTA Buffer. Irrelevant lanes between the panels were removed (C).

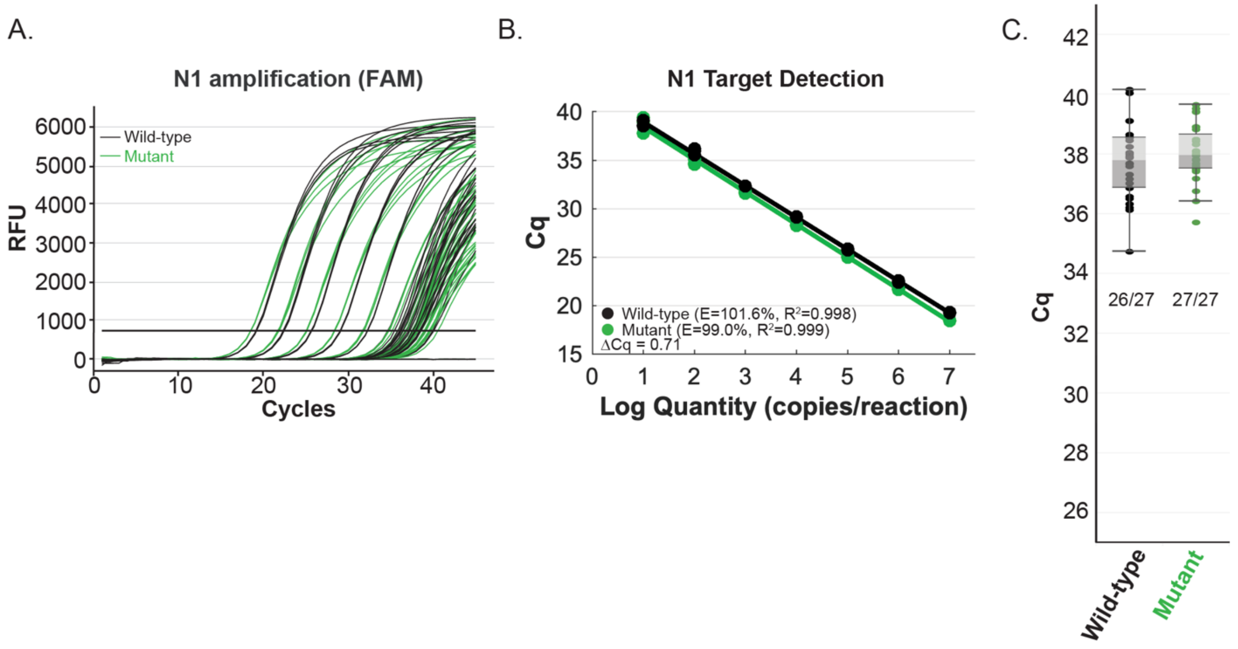

Supplemental Fig. 3. The SalivaDirect RT-qPCR protocol efficiently detects N gene IVT RNA carrying the Omicron mutation with the CDC 2019-nCoV_N1 primer-probe set. Amplification efficiency in A and B was evaluated in triplicate over a 7-log range (10^7-10 copies/reaction) of synthetic wild-type RNA (black) versus the Omicron mutant RNA (green). Detection sensitivity (C) was evaluated with 10 copies of RNA per reaction with 27 replicates per condition. All reactions tested with 10 copies of input RNA were detected by qPCR, except one wild-type sample amplified using the SalivaDirect RT-qPCR protocol.

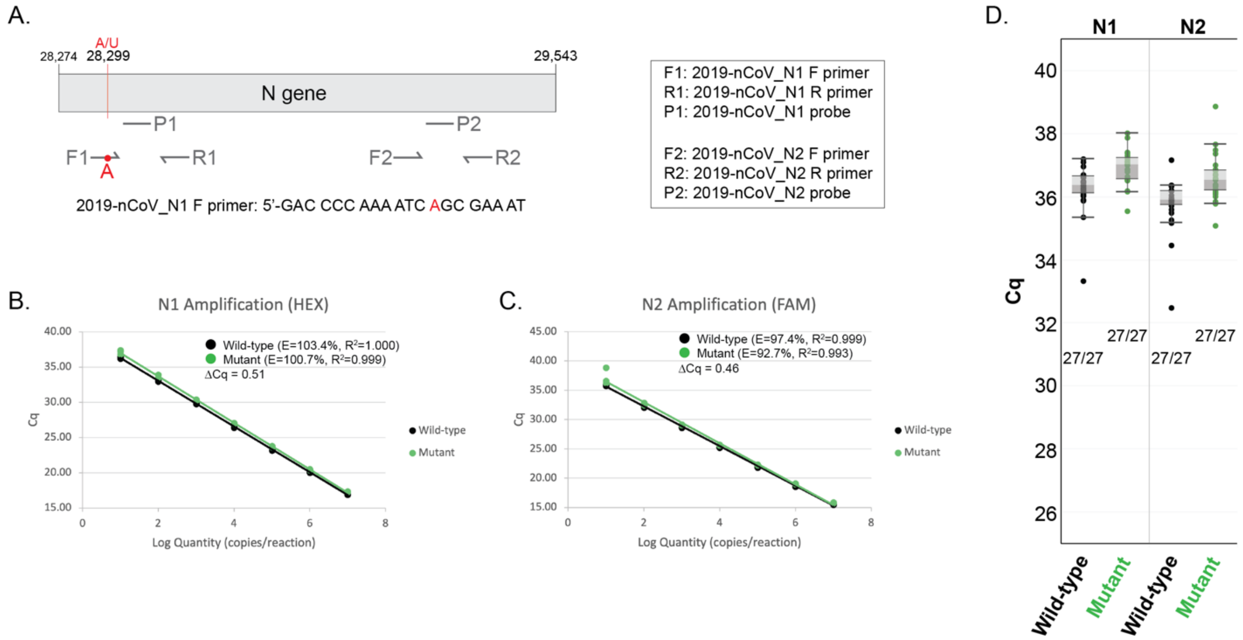

Supplemental Fig. 4. The NEB SARS-CoV-2 Multiplex Assay efficiently detects N gene IVT RNA carrying the AY43 (Delta) variant mutation with the CDC 2019-nCoV_N1 primer-probe set. A) Schematic representation of the two CDC primer-probe sets. Each set includes one forward primer (F), one reverse primer (R), and one fluorescent probe (P). The SARS-CoV-2 AY43 (Delta) variant has an A to U mutation (red) at position 28,299, which overlaps with the 2019-nCoV_N1 forward primer (F1) target sequence. Not drawn to scale. Amplification efficiency was evaluated at the N1 (B) and N2 (C) target sites in triplicate over a 7-log range (10^7-10 copies/reaction) of IVT wild-type RNA (black) versus the IVT AY43 mutant RNA (green). D) Detection sensitivity was evaluated with 10 copies of RNA per reaction with 27 replicates per condition. All reactions tested with 10 copies of input RNA were detected by qPCR.

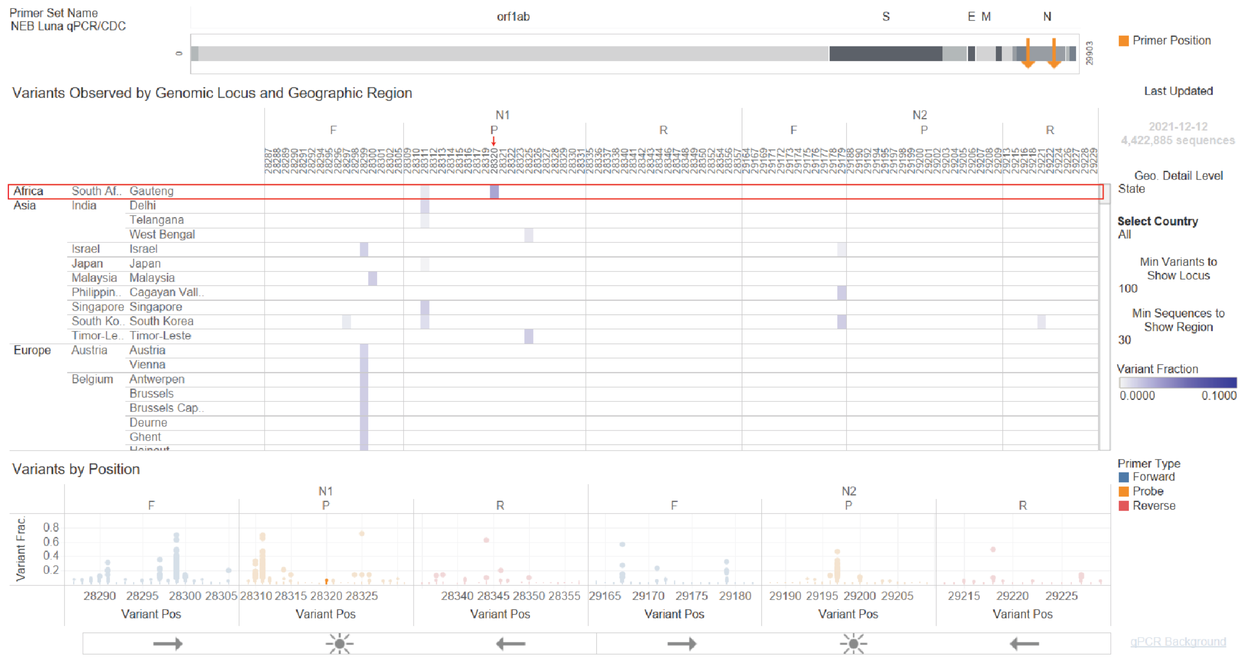

Supplemental Fig. 5. Second mutation at genetic position 28,320 overlaps with CDC 2019-nCoV probe target site. The mutation at genetic position 28,320 was identified using the Primer Monitor tool on 12/13/2021. The variants from Gauteng, South Africa and the mutation at position 28,320 are annotated with a red box and an arrow, respectively.

Supplemental Table 1. Primer Scheme Designs

Primer Scheme	Average Amplicon Size	Single- or Multi-reference-based Design?	Primer Set Sequence Release Date
ARTICv3	400 bp	Single	January 2020
Midnight-1200	1200 bp	Single	May 2020
ARTICv4	400 bp	Multiple (8)	June 2021
ARTICv4.1	400 bp	Multiple (9)	December 2021
VarSkip Short	560 bp	Multiple (>1,000,000)	September 2021
VarSkip Short v2	560 bp	Multiple (>1,000,000)	February 2022
VarSkip Long	1400 bp	Multiple (>1,000,000)	January 2022

Supplemental Table 2. N1 and N2 target detection from clinical samples

Variant	N1 target Cq	N2 target Cq	Cq(N2-N1)
Non-Omicron	30.14	34.57	4.43
Non-Omicron	28.6	26.9	-1.70
Non-Omicron	24.52	24.3	-0.22
Non-Omicron	23.24	22.62	-0.62
Non-Omicron	28.7	27.35	-1.35
Non-Omicron	24.35	22.84	-1.51
Non-Omicron	27.26	26.56	-0.70

Non-Omicron	18.3	18.07	-0.23
Non-Omicron	24.92	23.86	-1.06
Non-Omicron	25.21	25.07	-0.14
Non-Omicron	29.99	29.66	-0.33
Non-Omicron	20.61	20.12	-0.49
Non-Omicron	20.5	19.9	-0.60
Non-Omicron	22.61	21.99	-0.62
Non-Omicron	29.99	29.99	0.00
Non-Omicron	20.61	20.61	0.00
Non-Omicron	31.71	30.85	-0.86
Non-Omicron	32.62	31.67	-0.95
Non-Omicron	31.19	30.16	-1.03
Non-Omicron	31.74	31.56	-0.18
Non-Omicron	27.52	28.63	1.11
Non-Omicron	18.19	18.48	0.29
Omicron	24.15	23.13	-1.02
Omicron	35.34	35.08	-0.26
Omicron	32.45	31.47	-0.99
Omicron	30.65	29.79	-0.86
Omicron	25.44	24.51	-0.92

Omicron	29.48	28.54	-0.94
Omicron	31.73	31.07	-0.67
Omicron	30.34	29.41	-0.93
Omicron	28.98	28.14	-0.84
Omicron	23.57	22.54	-1.03
Omicron	31.11	30.22	-0.89
Omicron	19.83	18.69	-1.14
Omicron	31.32	30.44	-0.88
Omicron	34.23	33.64	-0.59
Omicron	28.98	28.06	-0.92
Omicron	27.43	26.47	-0.96
Omicron	26.67	25.77	-0.90
Omicron	18.20	17.15	-1.05
Omicron	24.38	23.34	-1.03
Omicron	31.56	30.83	-0.72
Omicron	23.07	22.07	-1.00
Omicron	22.04	20.98	-1.06
Omicron	23.18	22.18	-1.00
Omicron	28.08	27.06	-1.02
Omicron	24.04	23.01	-1.03

Omicron	25.18	24.15	-1.03
Omicron	24.53	23.45	-1.08
Omicron	25.96	24.9	-1.06
Omicron	20.05	19	-1.05
Omicron	33.13	32.19	-0.94
Omicron	32.47	31.87	-0.61
Omicron	23.16	22.34	-0.82
Omicron	28.22	27.51	-0.71
Omicron	19.87	19.04	-0.82
Omicron	31.37	30.75	-0.62
Omicron	28.48	27.79	-0.69
Omicron	23.56	22.77	-0.79
Omicron	27.29	26.58	-0.71
Omicron	22.79	22.01	-0.78
Omicron	28.46	27.76	-0.70
Omicron	29.59	29.01	-0.58
Omicron	29.80	29.21	-0.59
Omicron	25.43	24.80	-0.63
Omicron	34.32	33.59	-0.73
Omicron	27.53	26.89	-0.64

Omicron	28.59	27.98	-0.61
Omicron	17.04	16.07	-0.97
Omicron	28.78	28.11	-0.67
Omicron	20.91	20.08	-0.83
Omicron	30.02	29.23	-0.79
Omicron	31.63	30.88	-0.75
Omicron	32.47	31.8	-0.67
Omicron	27.82	26.96	-0.86
Omicron	32.29	31.52	-0.77
Omicron	26.35	25.44	-0.91
Omicron	29.96	29.09	-0.87
Omicron	28.19	27.44	-0.75
Omicron	34.08	33.43	-0.65
Omicron	31.44	30.65	-0.79
Omicron	30.35	29.53	-0.82
