# Supplementary material for: Overcoming variant mutation-related impacts on viral sequencing and detection methodologies
Source: Front Med (Lausanne). 2022 Oct 28;9:989913. doi: 10.3389/fmed.2022.989913 (PMC9650041; doi:10.3389/fmed.2022.989913)
Supplement: Supplementary file 2 [file Data_Sheet_2.PDF]

The datasets presented in this study can be found in online repositories. The names of the repository/repositories and accession number(s) can be found below:

https://www.ncbi.nlm.nih.gov/

PRJNA717314

OP354513

OP354514

OP354515

OP354516

OP354517

OP354518

OP354519

OP317643

OP317644

OP317645

OP317646

OP317647

OP317648

OP317649

OP317650

OP317651

OP317652

OP317653

OP317654

OP317655

OP317656

OP317657

OP317658

OP317659

OP317660

OP317661

OP317662

OP317663

OP317664

OP317665

OP317666

OP317667

OP317668

OP317669

OP317670

OP317671

OP317672

OP317673

OP317674

OP317675

OP317676

OP317677

OP317678

OP317679

OP317680

OP317681

OP317682

OP317683

OP317684

OP317685

OP317686

OP317687

OP317688

OP317689

OP317690

OP317691

OP317692

ON849049

ON849050

ON849051

ON849052

ON849053

ON798858

ON798859

ON798860

ON798861

ON798862

ON798863

ON798864

ON798865

ON798866

ON798867

ON798868

ON798869

ON798870

ON798871

ON798872

ON798873

ON798874

ON798875

ON798876

ON798877

ON798878

ON798879

ON798880

ON798881

ON798882

ON798883

ON798884

ON798885

ON798886

ON798887

ON798888

ON798889

ON798890

ON798891

ON798892

ON798893

ON798894

ON798895

ON798896

ON798897

ON798898

ON798899

ON798900

ON798901

ON798902

ON798903

ON798904

ON798905

ON798906

ON798907

ON798908

ON798909

ON798910

ON798911

ON798912

ON798913

ON798914

ON798915

ON798916

ON798917

ON798918

ON798919

ON798920

ON798921

ON798922

ON798923

ON798924

ON798925

ON798926

ON798927

ON798928

ON798929

ON798930

ON798931

ON798932

ON798933

ON798934

ON798935

ON798936

ON798937

ON798938

ON798939

ON798940

ON798941

ON798942

ON798943

ON798944

ON798945

ON798946

ON798947

ON798948

ON798949

ON798950

ON798951

ON798952

ON798953

ON798954

ON798955

ON798956

ON798957

ON798958

ON798959

ON798960

ON798961

ON798962

ON798963

ON798964

ON798965

ON798966

ON798967

ON798968

ON798969

ON798970

ON798971

ON798972

ON798973

ON798974

ON798975

ON798976

ON798977

ON798978

ON798979

ON798980

ON798981

ON798982

ON798983

ON798984

ON798985

ON798986

ON798987

ON798988

ON798989

ON798990

ON798991

ON798992

ON798993

ON798994

ON798995

ON798996

ON798997

ON798998

ON798999

OM250086

OM250087

OM250088

OM250089

OM250090

OM250091

OM250092

OM250093

OM250094

OM250095

OM250096

OM250097

OM250098

OM250099

OM250100

OM250101

OM250102

OM131540

OM131541

OM131542

OM131543

OM019194

OM019195

OM019196

OL958420

OL958421

OL958422

OL958423

OL453192

OL453193

OL453194

OL305825

OL305826

OK284597

OK185935

OK185936

OK185937

OK185938

MZ710932

MZ710933

MW819849
